# Single-Cell Transcriptomic Analysis of Rat Urethral Tissue Following Simulated Childbirth Injury and Microenergy Acoustic Pulse Therapy

**DOI:** 10.3390/ijms27219833

**Published:** 2026-11-03

**Authors:** Guiting Lin, Liangyu Zhao, Emily Xing, Tian Bai, Hao Li, Yuan Tang, Thomas Cao, Guifang Wang, Yuxin Tang, Tom F. Lue

**Affiliations:** 1 Knuppe Molecular Urology Laboratory, Department of Urology, School of Medicine, University of California, San Francisco, CA 94143, USA; guiting.lin@ucsf.edu (G.L.); zhaoly37@mail.sysu.edu.cn (L.Z.); andyty1985@163.com (Y.T.); tangyx36@mail.sysu.edu.cn (Y.T.); 2 Department of Urology, Department of Interventional Medicine, and Guangdong Provincial Key Laboratory of Biomedical Imaging, The Fifth Affiliated Hospital, Sun Yat-sen University, Zhuhai 519000, China; 3 Department of Urology, Tongji Hospital, Tongji Medical College, Huazhong University of Science and Technology, Wuhan 430030, China; 4 Andrology Center, Peking University First Hospital, Beijing 100034, China

**Keywords:** stress urinary incontinence, microenergy acoustic pulse, single-cell RNA sequencing, urethral repair, aging, cell-cell communication

## Abstract

Microenergy acoustic pulse (MAP) therapy has been associated with functional and structural improvement after childbirth-related urethral injury, but its multicellular effects remain incompletely defined. We explored treatment- and injury-associated differences in the urethral microenvironment using single-cell RNA sequencing. Eight female Sprague–Dawley rats were assigned to sham, vaginal balloon dilation plus ovariectomy (VBDO), VBDO plus beta-aminopropionitrile (BAPN), or VBDO plus BAPN and MAP (MAP) groups, with one 24-week-old and one 48-week-old rat per group. Urethral tissues were analyzed for cell composition, differential gene expression, Gene Ontology enrichment, stem/progenitor features, senescence-associated secretory phenotype activity, and CellChat-inferred intercellular communication. A balanced single-cell dataset comprising 23,128 cells was used for downstream comparative analyses and resolved 19 annotated cell-type/state categories. Across the injury- and age-associated experimental conditions, epithelial, vascular, stromal, immune, and muscle compartments showed distinct transcriptional patterns, whereas MAP-treated samples showed a distinct cellular distribution; a separate histological cohort showed higher urethral muscle content. MAP-treated samples also showed differences in CellChat-inferred signaling activity in selected extracellular-matrix, adhesion, vascular, developmental, and neural/neuromuscular pathways, including fibronectin 1 (FN1), collagen (COLLAGEN), angiopoietin (ANGPT), vascular endothelial growth factor (VEGF), Wingless/Integrated (WNT), Notch (NOTCH), reelin (RELN), Slit guidance ligand (SLIT), neural cell adhesion molecule (NCAM), and neurexin (NRXN). Exploratory enrichment analysis identified treatment-associated differences in translation, oxidative metabolism, stress adaptation, synaptic-organization processes, progenitor-associated transcriptional states, and senescence-related programs. The treatment-split marker manifold approximation and projection (UMAPs) are descriptive and do not provide evidence of increased muscle-cell number; the reported difference in muscle-associated staining content derives from a separate animal-level histological analysis. Overall, the findings are descriptive and hypothesis-generating and require validation in independently replicated cohorts.

## 1. Introduction

Stress urinary incontinence (SUI) is defined as involuntary urine leakage during physical activity, exertion, coughing, or sneezing [1,2]. Vaginal delivery and aging are major risk factors for SUI, and the combination of childbirth-related tissue injury, estrogen deprivation, and age-related loss of regenerative capacity may contribute to persistent urethral dysfunction [3,4,5,6]. Current treatment is centered on pelvic floor muscle training, urethral bulking, and surgery; however, therapies that directly restore the damaged urethral microenvironment remain limited [7,8]. Urethral continence depends on coordinated interactions among smooth muscle, the external striated urethral sphincter, connective tissue, vasculature, peripheral nerves, immune cells, and the urothelial barrier [9,10,11]. Vaginal distension models show that simulated childbirth injury can disrupt sphincter structure and extracellular matrix integrity, whereas ovariectomy and inhibition of collagen crosslinking can further aggravate tissue damage [12,13]. Our previous single-cell study further suggested that aging redirects urethral repair from coordinated regeneration toward muscle loss, denervation-related transcriptional changes, fibroblast activation, inflammation, extracellular matrix deposition, and urothelial senescence.

Microenergy acoustic pulse (MAP) is a low-intensity mechanical stimulation therapy designed to activate endogenous tissue repair without thermal ablation. In animal models of SUI, MAP improves leak-point pressure and restores pelvic floor and urethral muscle structure, accompanied by activation of resident progenitor cells and enhancement of repair-related signaling [14]. However, the effects of MAP on individual urethral cell populations and multicellular communication networks remain unclear.

Single-cell RNA sequencing (scRNA-seq) can resolve rare cell states in complex tissues and infer ligand-receptor communication within the urethral microenvironment [15,16,17,18]. This exploratory study integrated single-cell data from eight animals representing aging, injury, fibrosis-prone injury, and MAP treatment states. We examined treatment-associated differences in cellular community structure, inferred intercellular communication networks, and regenerative, senescence, and stress-associated transcriptional states. Given the limited biological replication, these analyses were considered descriptive and hypothesis-generating rather than definitive evidence of treatment mechanisms.

## 2. Results

### 2.1. The Single-Cell Atlas Resolves a Complex Multicellular Urethral Ecosystem

**Figure 1:**
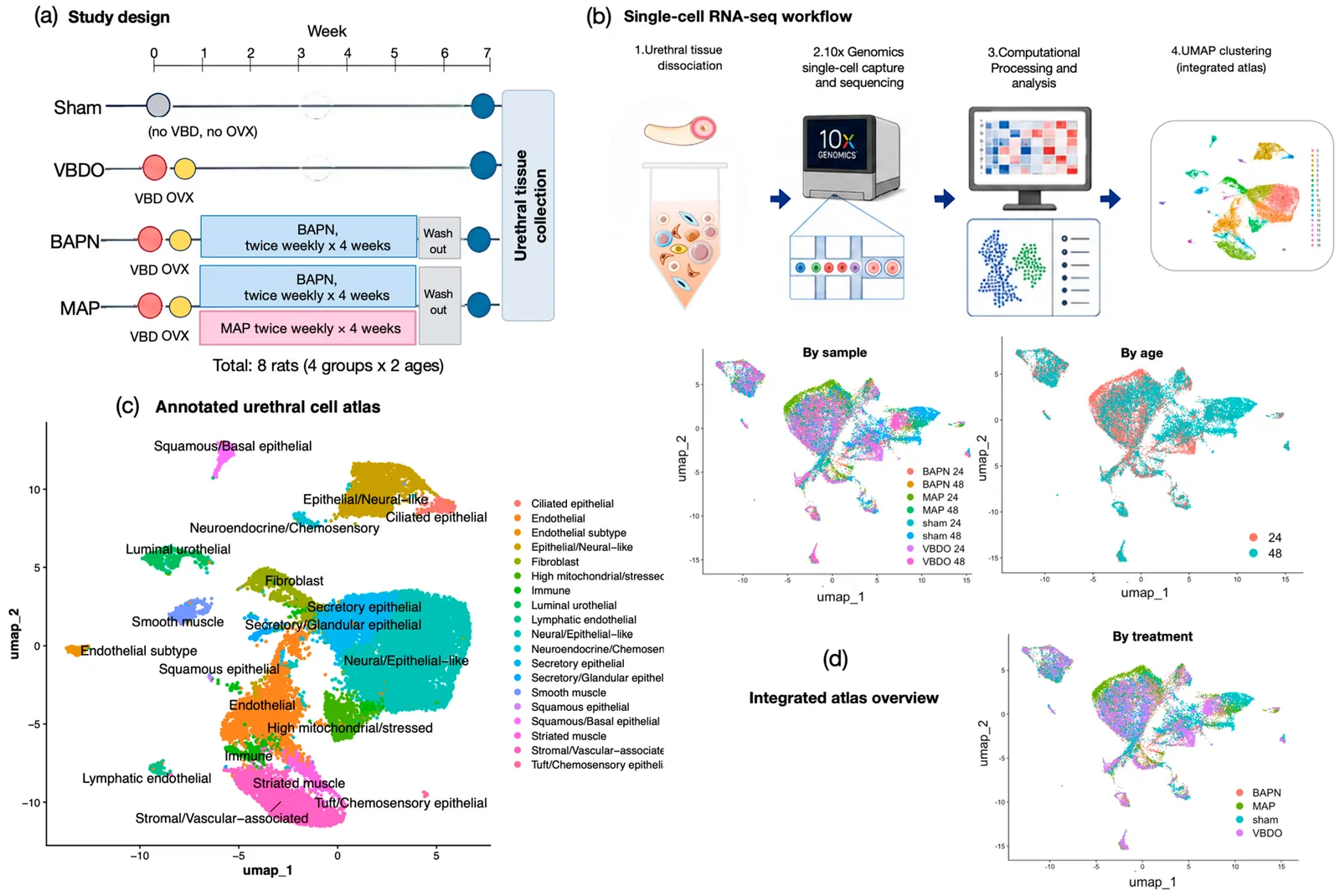
Experimental design, single-cell RNA-sequencing workflow, and integrated urethral cell atlas. (**a**) Study design and experimental timeline. Female Sprague–Dawley rats at 24 and 48 weeks of age were assigned to Sham, VBDO, VBDO + BAPN, and VBDO + BAPN + MAP groups, with one animal per age-by-treatment condition (total *n* = 8). VBD was performed at Week 0, bilateral ovariectomy at Week 1, and BAPN treatment was administered twice weekly for 4 weeks beginning at Week 2. MAP treatment was given concurrently with BAPN in the MAP group. After a 1-week washout, urethral tissues were collected at Week 7. (**b**) Single-cell RNA-sequencing workflow from urethral tissue dissociation and 10× Genomics sequencing to computational processing, integration, and UMAP clustering. (**c**) Annotated UMAP of the integrated urethral single-cell atlas showing the major transcriptionally defined cell populations. (**d**) Integrated UMAPs colored by individual sample, age, and treatment group to show the contribution of each experimental condition to the atlas. A detailed week-by-week experimental timeline is provided in Supplementary Figure S1.

UMAP visualization showed broad integration across individual samples, treatment groups, and ages while preserving biologically distinct cell populations. Integrated analysis identified 19 annotated cell-type/state categories spanning ciliated, luminal, secretory, glandular, squamous, basal, tuft/chemosensory, neuroendocrine, and epithelial/neural-like states, together with endothelial, lymphatic endothelial, fibroblast, stromal/vascular-associated, immune, smooth muscle, striated muscle, and high-mitochondrial/stressed populations. Cell-type identities were assigned using representative canonical marker genes, as described in the Methods. To reduce unequal representation caused by differences in cell recovery, a balanced subset of 23,128 cells was retained after sample-level downsampling for comparative downstream analyses (Table S1; Section 4). These findings establish the urethra as a multicellular tissue ecosystem rather than an isolated muscular conduit (Figure 1a–d).

### 2.2. Transcriptional Differences Across Injury- and Age-Associated Experimental Conditions

Comparison of age and injury conditions indicated a shift from coordinated homeostasis toward a maladaptive repair state. Muscle populations showed reduced regenerative and neuron-projection-associated programs, consistent with impaired neuromuscular integrity. Fibroblast and stromal populations were enriched for extracellular-structure organization, growth-factor responses, and wound-remodeling programs, whereas immune populations showed inflammatory activation. Differentiated urothelial features were reduced, and senescence-associated activity increased in selected epithelial states. Collectively, the experimental conditions representing childbirth-like injury, estrogen deprivation, fibrosis-prone remodeling, and different ages were associated with coordinated differences involving muscle, matrix, vasculature, immunity, and epithelial barrier function (Figure 1d). Given the limited biological replication, the independent contributions of age and treatment cannot be resolved.

### 2.3. MAP-Associated Cell-State Patterns and Independently Quantified Urethral Muscle Content

**Figure 2:**
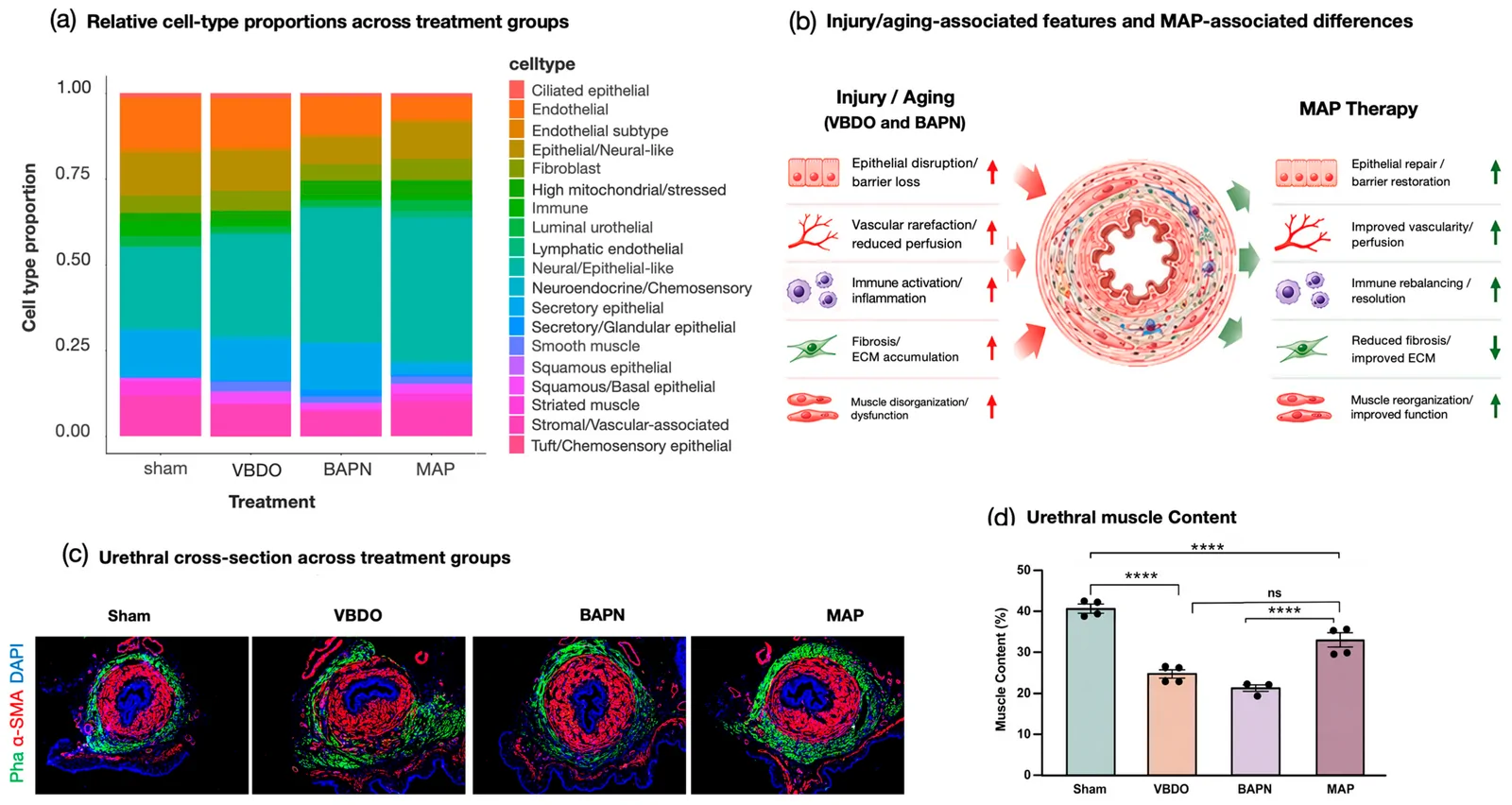
Treatment-associated differences in the injured urethral microenvironment and urethral muscle content in a separate histological cohort. (**a**) Relative proportions of major urethral cell populations identified by single-cell RNA sequencing in the Sham, VBDO, BAPN, and MAP groups. Relative cell-type distributions differed descriptively among groups; MAP-treated samples differed from BAPN and were not identical to the Sham profile. (**b**) Schematic summary of injury/aging-associated features and MAP-associated differences in the urethral ecosystem. (**c**) Representative transverse urethral sections stained with phalloidin (F-actin; green), α-smooth muscle actin (α-SMA; red), and DAPI (blue) in the indicated groups. This histological cohort consisted of 16 additional 24-week-old female Sprague–Dawley rats that were not included in the scRNA-seq cohort. Animals were assigned to Sham (*n* = 4), VBDO (*n* = 4), BAPN (*n* = 4), and MAP (*n* = 4) groups. (**d**) Quantification of muscle-associated staining content in the urethral cross-sections. Each dot represents one independent animal (*n* = 4 per group), and bars show mean ± SEM. Statistical analysis was performed using one-way ANOVA followed by Tukey’s multiple-comparisons test. Adjusted *p*-values are indicated for the specified pairwise comparisons; **** *p* < 0.0001; ns, not significant. Muscle content (%) was calculated from ImageJ 1.54r-measured positive staining areas as [(α-SMA-positive red area + phalloidin/F-actin-positive green area)/total urethral tissue region-of-interest area] × 100. This area-based measurement is not a direct measure of muscle-cell number.

**Figure 3:**
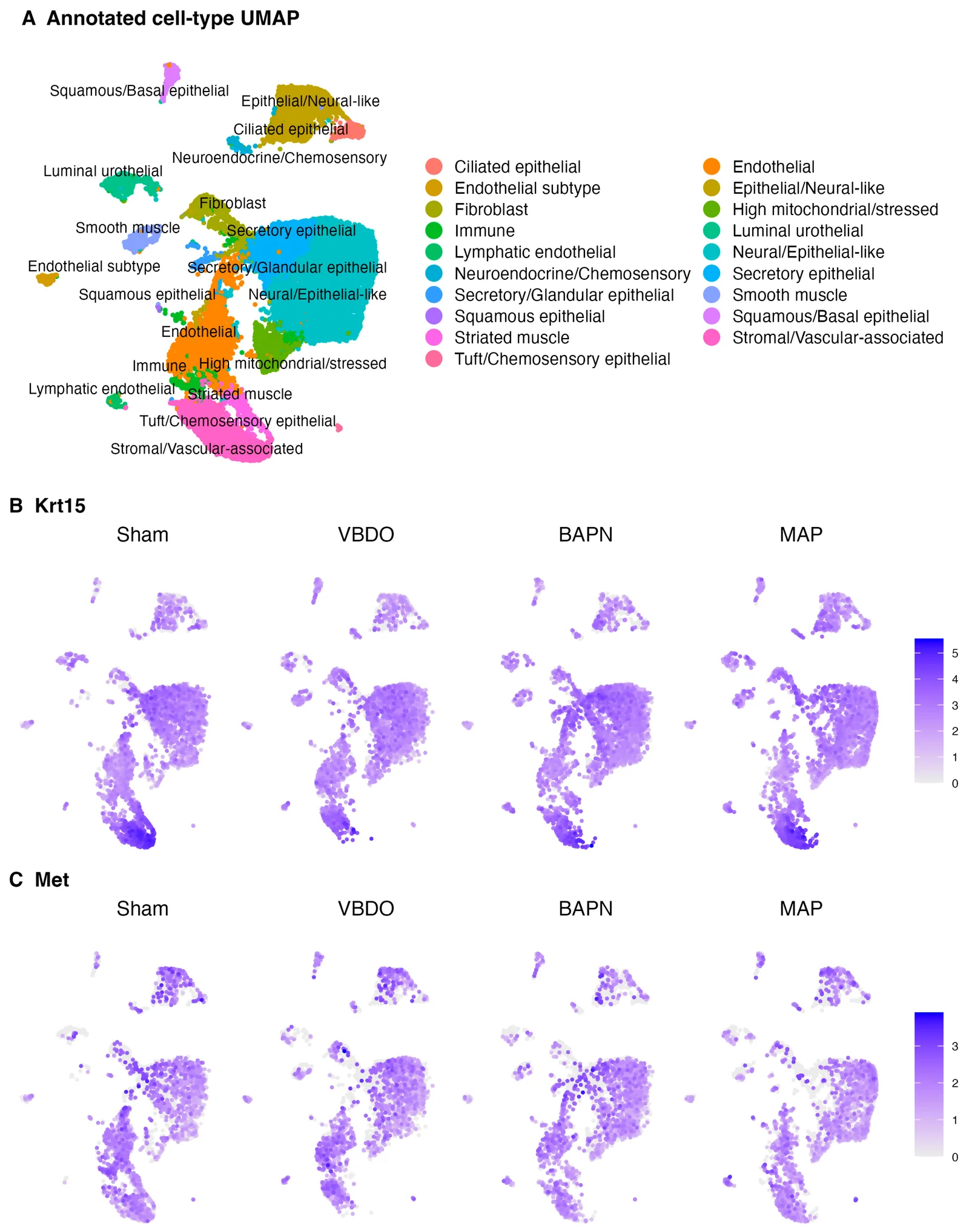
Cell-type atlas and treatment-specific expression of Krt15 and Met. (**A**) UMAP visualization of the integrated urethral single-cell transcriptomic dataset, with cells annotated according to major cell-type identities. (**B**) Treatment-split UMAP feature plots showing Krt15 expression in Sham, VBDO, VBDO + BAPN, and VBDO + BAPN + MAP groups. (**C**) Treatment-split UMAP feature plots showing Met expression across the same four experimental conditions. For each gene, the same UMAP coordinates and expression scale were used across treatment groups to facilitate direct visual comparison. These plots are presented as descriptive visualizations of cell-level expression patterns. For visualization, cells from the 24- and 48-week animals were displayed together within each treatment group; these plots do not constitute independent treatment-level biological replication.

The relative proportions of annotated cell states varied descriptively among Sham, VBDO, BAPN, and MAP samples (Figure 2a). However, the single-cell dataset contains only one animal per age-by-treatment condition, and these relative cell-recovery proportions cannot establish a MAP-induced increase in the absolute number of smooth or striated muscle cells. The treatment-split UMAPs now presented in the main text (Figure 3) map Krt15 and Met expression onto the common cell atlas. Their broadly similar distributions across conditions should not be interpreted as evidence of muscle-cell expansion; moreover, Krt15 and Met are not muscle-mass measurements. In contrast, the independent histological cohort (four 24-week-old animals per group) provides a separate tissue-level readout (Figure 2c,d): representative transverse sections show phalloidin-positive F-actin structures (green) and α-SMA-positive smooth muscle (red), while the animal-level quantification in Figure 2d shows higher measured urethral muscle-associated staining content in MAP than BAPN animals. The relevant comparison should therefore be assessed principally in the quantified Figure 2d, together with the corresponding MAP and BAPN sections in Figure 2c, rather than in the UMAP feature plots. This histological measurement reflects muscle-associated staining content and does not, by itself, measure new muscle-cell formation or distinguish smooth- from striated-muscle contributions.

### 2.4. MAP Treatment-Associated Differences in Inferred Signaling and Biological Programs

**Figure 4:**
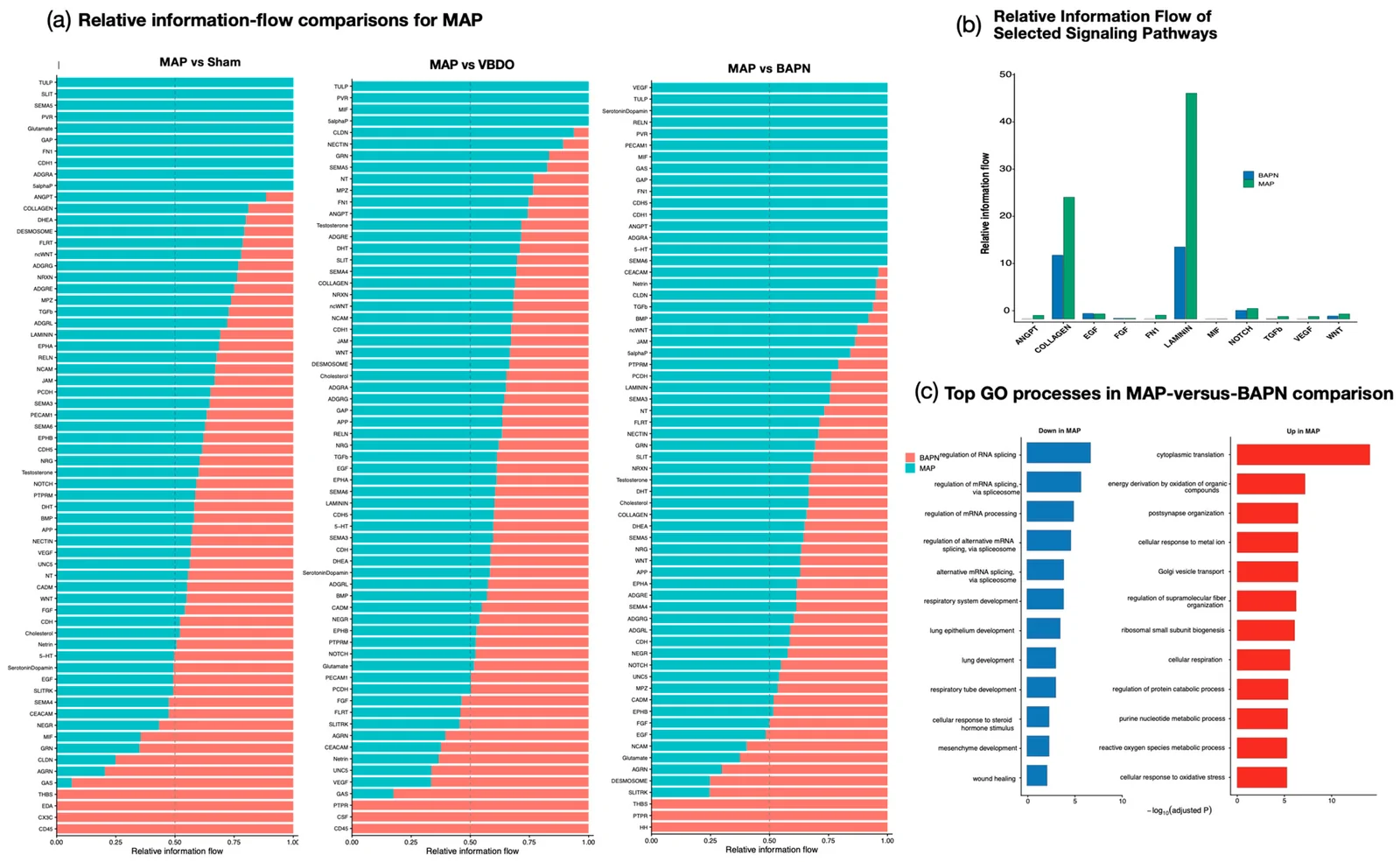
MAP treatment-associated differences in CellChat-inferred intercellular communication and biological processes in urethral tissues. (**a**) CellChat-based comparison of relative information flow between MAP and the sham, VBDO, and BAPN groups. Each horizontal stacked bar represents a signaling pathway, with the relative contribution of MAP and the corresponding comparator normalized to a total of 1. Pathways with a greater MAP-associated fraction indicate relatively higher inferred signaling activity in MAP-treated samples, whereas pathways dominated by the comparator indicate relatively lower inferred activity in MAP-treated samples. (**b**) Relative information flow of representative signaling pathways in the MAP and BAPN groups. (**c**) Exploratory Gene Ontology biological-process enrichment analysis based on the cell-level MAP-versus-BAPN comparison. Blue bars show processes associated with genes showing lower expression in MAP-treated samples, whereas red bars show processes associated with genes showing higher expression in MAP-treated samples. Bar length represents the −log10-adjusted *p*-value from the exploratory cell-level analysis and does not represent treatment-group inference across independent animals.

CellChat-based information-flow comparisons showed differences in inferred signaling profiles between MAP-treated samples and sham, VBDO, and BAPN samples (Figure 4a). Relatively higher inferred activity in MAP-treated samples involved extracellular-matrix and basement-membrane pathways, cell adhesion and junctional signaling, vascular remodeling, developmental regulation, and neural communication. Representative pathways included COLLAGEN, LAMININ, FN1, ANGPT, VEGF, PECAM1, CDH5, CLDN, NECTIN, JAM, WNT/ncWNT, TGFβ, BMP, RELN, SLIT, NCAM, NRXN, MPZ, and semaphorin signaling. Treatment-specific and lineage-specific expression of selected repair-associated candidate genes is shown in main Figure 3, Figures S2 and S3. The cell-type distribution of representative marker-gene and pathway-associated genes is shown in Supplementary Figures S4 and S5. In the MAP-versus-BAPN comparison, LAMININ- and FN1-associated information flow was particularly prominent, consistent with relatively greater inferred cell-matrix and basement-membrane communication (Figure 4b). Exploratory Gene Ontology analysis of the cell-level comparison showed that genes with higher expression in MAP-treated samples were associated with cytoplasmic translation, oxidative energy metabolism, cellular respiration, Golgi-mediated transport, reactive oxygen species handling, postsynaptic organization, synapse regulation, and apoptotic-signal regulation. Conversely, genes with lower expression in MAP-treated samples were associated with RNA processing, steroid-hormone responses, mesenchymal development, developmental growth, and persistent wound-remodeling programs. These enrichment results are descriptive and hypothesis-generating because adjusted *p*-values derive from cell-level exploratory analyses rather than independent animals. Together, these descriptive differences involved transcriptional features related to prolonged-injury signaling, metabolic competence, stress adaptation, and structural organization (Figure 4c).

### 2.5. MAP Treatment-Associated Differences in Epithelial-Stromal-Vascular-Muscle Communication

**Figure 5:**
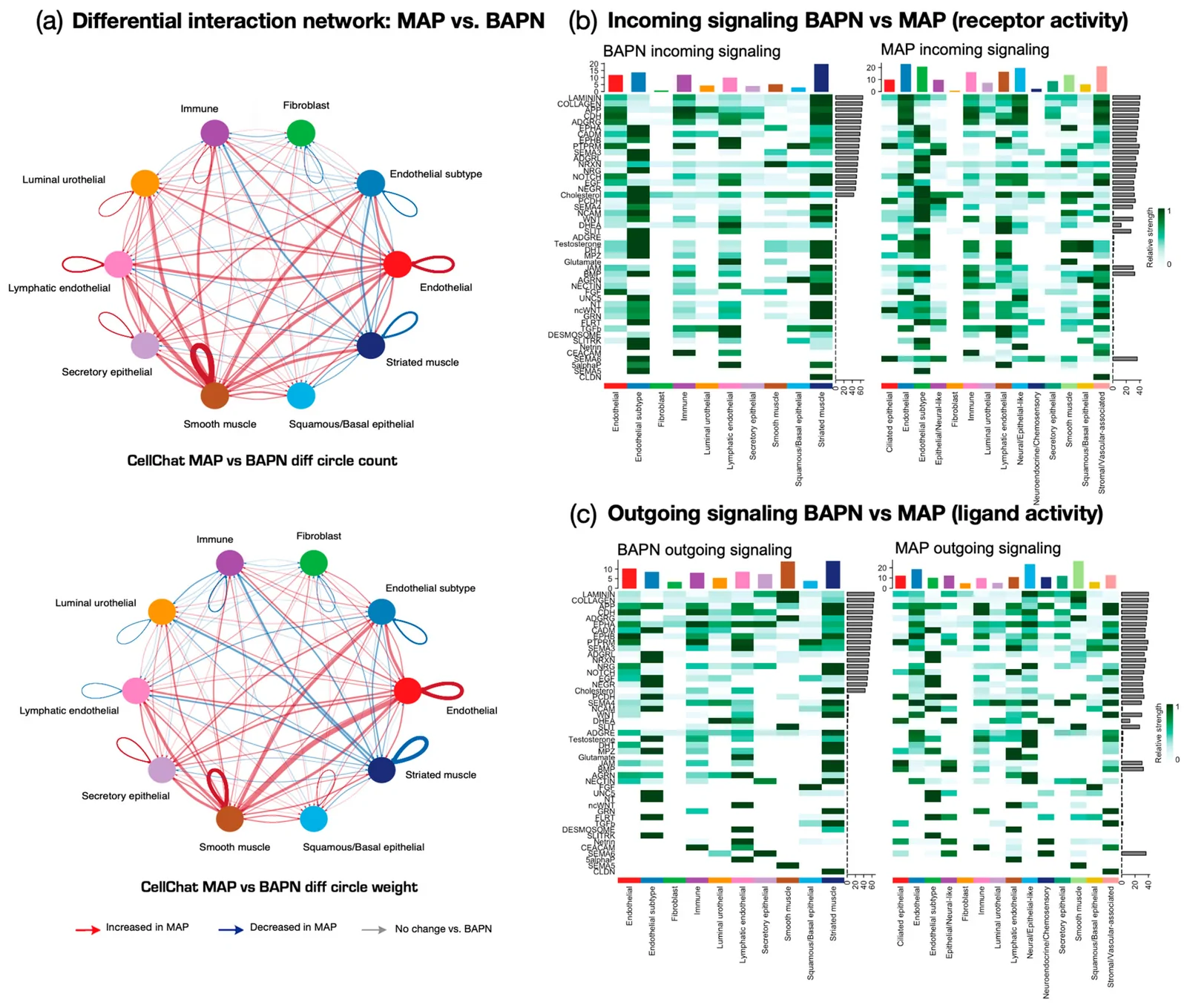
CellChat analysis identifies treatment-associated differences in predicted intercellular communication between MAP and BAPN groups. (**a**) Differential cell–cell interaction networks comparing the MAP and BAPN groups. The upper circle plot shows differences in the number of predicted ligand–receptor interactions in the MAP-versus-BAPN comparison, whereas the lower plot shows differences in overall interaction strength in the MAP-versus-BAPN comparison. Nodes represent major urethral cell populations, and node colors indicate cell identity. Red edges indicate interactions with greater inferred activity in MAP-treated samples, blue edges indicate interactions with lower inferred activity in MAP-treated samples, and gray edges indicate relatively unchanged inferred activity compared with BAPN samples. Edge thickness is proportional to the magnitude of the difference. (**b**) Heatmaps of incoming signaling activity, reflecting receptor-associated signaling received by each cell population in the BAPN and MAP groups. Rows represent signaling pathways and columns represent recipient cell types. Darker green indicates greater relative incoming signaling activity. (**c**) Heatmaps of outgoing signaling activity, reflecting ligand-associated signaling transmitted by each cell population in the BAPN and MAP groups. Rows represent signaling pathways and columns represent sender cell types. Darker green indicates greater relative outgoing signaling activity.

Differential CellChat networks comparing MAP with BAPN showed broad differences in both the number and aggregate strength of predicted ligand-receptor interactions (Figure 5a). The largest network differences involved endothelial and lymphatic endothelial cells, fibroblasts, immune cells, luminal and secretory epithelial populations, and smooth and striated muscle. Incoming-signaling heatmaps showed differences in receptor-associated signals received by multiple cell populations, including muscle compartments, whereas outgoing-signaling heatmaps showed parallel differences in ligand-associated signals transmitted to surrounding vascular, immune, epithelial, and matrix-associated cells (Figure 5b,c) (enlarged versions in Figures S6 and S7). The predicted network differences were distributed across multiple sender and receiver populations rather than being dominated by a single cell type or signaling pathway, indicating broad treatment-associated differences in bidirectional communication across the urethral microenvironment.

### 2.6. MAP-Associated Shifts in Progenitor-Associated Transcriptional States

**Figure 6:**
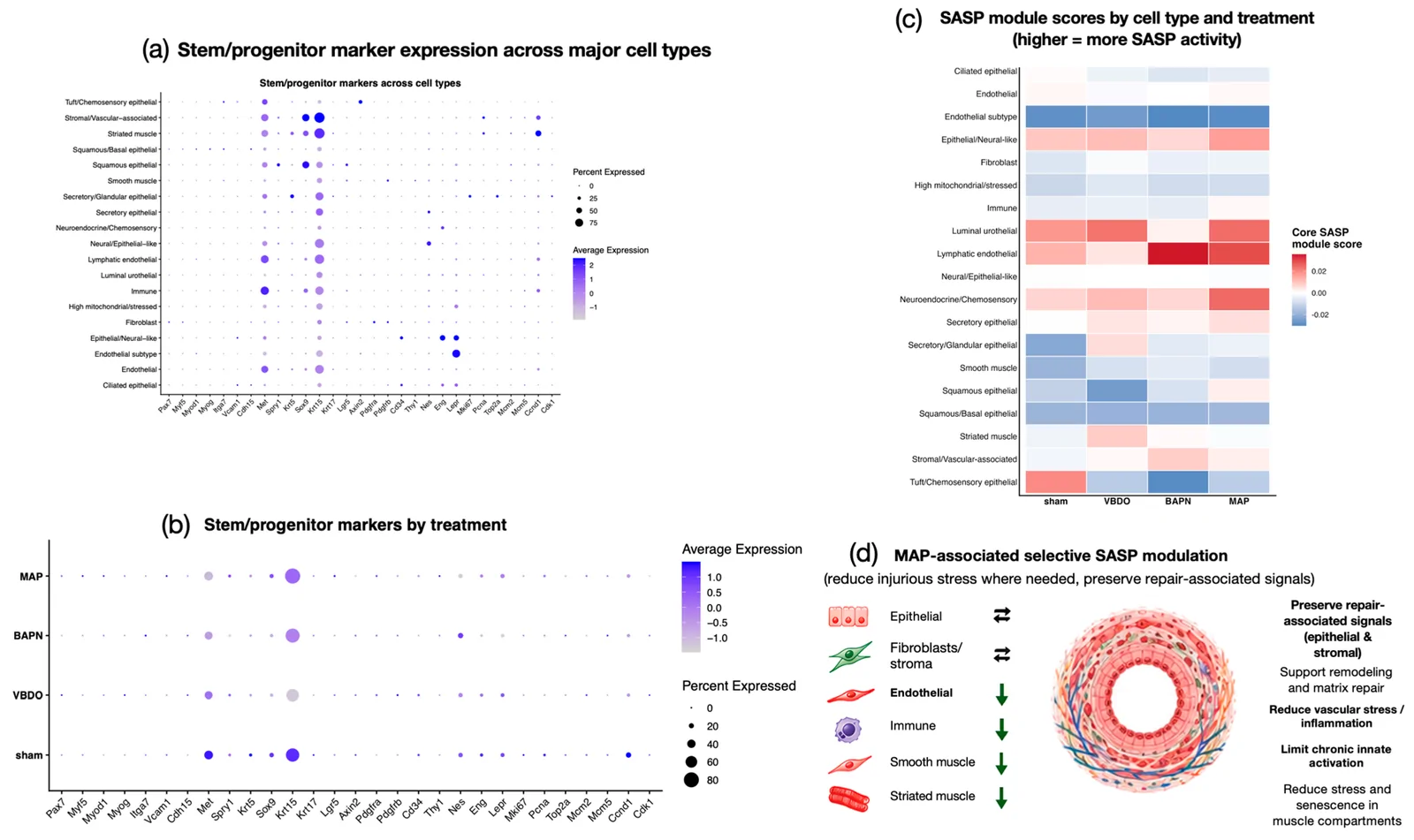
Stem/progenitor cell signatures and selective modulation of senescence-associated secretory phenotype activity following MAP treatment. (**a**) Dot plot showing the expression of representative stem/progenitor-associated markers across major urethral cell populations. Dot size indicates the percentage of cells expressing each gene, and color intensity represents the average scaled expression level. (**b**) Dot plot comparing stem/progenitor marker expression among treatment groups. Dot size represents the percentage of expressing cells, whereas color intensity indicates average expression. (**c**) Heatmap of senescence-associated secretory phenotype (SASP) module scores across cell types and treatment groups. Red indicates relatively increased SASP activity, whereas blue indicates reduced SASP activity. MAP-treated samples showed a cell type–selective pattern rather than a uniform suppression of SASP signaling. (**d**) Schematic summary of MAP-associated selective SASP modulation within the urethral tissue microenvironment.

Stem/progenitor-associated markers displayed lineage-restricted expression across the urethral atlas (Figure 6a). Epithelial progenitor-like populations expressed markers including Krt15 and Sox9, muscle-associated cells expressed Itga7, and vascular/stromal repair populations expressed Cd34 and Met. Figure 6b provides descriptive treatment-group dot-plot summaries of progenitor-associated marker expression. Treatment-split UMAPs for Krt15 and Met are shown in Figure 3B,C; additional UMAPs for Sox9, Itga7, and Cd34 and lineage-specific violin/dot plots are provided in Supplementary Figures S2 and S3. These observations are consistent with differences in progenitor-associated transcriptional states but do not establish lineage expansion, progenitor reactivation, or regeneration. The displayed marker expression and module scores describe transcriptional features only and do not demonstrate functional activation of progenitor cells.

### 2.7. MAP Treatment-Associated Differences in SASP Activity Across Urethral Lineages

SASP module scores varied substantially by cell type and treatment (Figure 6c). Relatively high activity occurred in selected squamous/basal epithelial, fibroblast, epithelial/neural-like, neuroendocrine/chemosensory, and muscle-associated populations, whereas several endothelial, immune, and other epithelial states showed lower scores. MAP was associated with lower SASP-associated module scores in selected vascular, immune, smooth-muscle, and striated-muscle populations, whereas epithelial and stromal populations showed a different pattern. The schematic model summarizes this lineage-specific balance between limiting chronic injury signaling and maintaining repair-associated communication (Figure 6d).

## 3. Discussion

### 3.1. MAP Treatment-Associated Remodeling of the Urethral Ecosystem

For decades, stress urinary incontinence (SUI) has often been viewed as a relatively simple mechanical failure, primarily attributed to lax urethral support or insufficient sphincter contraction, with treatment therefore focused on tightening, supporting, or repairing damaged tissues [2]. The urethra is not a passive conduit composed of a single muscular structure; rather, it is a dynamic tissue ecosystem maintained by epithelial, vascular, immune, stromal, neural, and muscle cells. SUI, particularly when associated with aging and childbirth injury, may therefore represent widespread dysfunction of this multicellular ecosystem rather than an isolated mechanical failure of muscle.

A key distinction is between the transcriptomic atlas and the independent tissue-level histology. The feature UMAPs visualize expression locations, not absolute cell counts. Although the histological cohort shows greater muscle-associated staining in the MAP group than in injured comparators (Figure 2c,d), this does not independently establish myogenesis, expansion of muscle cell number, or a causal explanation for the transcriptomic patterns.

In this study, scRNA-seq captured active transcriptional programs in individual cells, and UMAP grouped cells with similar expression profiles into distinct clusters, enabling construction of a high-resolution cellular atlas of the rat urethra. The integrated analysis identified transcriptionally distinct populations spanning epithelial, endothelial, lymphatic endothelial, immune, fibroblast, stromal/vascular-associated, smooth muscle, striated muscle, neuroendocrine, and stressed cell states.

The present data suggest several levels at which MAP-treated samples differ from injured comparators. First, cell-type composition showed treatment-associated redistribution. Second, CellChat analysis identified broad differences in predicted incoming and outgoing signaling across epithelial, endothelial, lymphatic endothelial, immune, fibroblast, and muscle populations; these predictions do not establish functional communication. Third, progenitor-associated and SASP-associated transcriptional states varied among treatment groups in a cell-type-dependent manner. These observations are descriptive and should be regarded as hypotheses for future studies with independent biological replication and orthogonal validation.

The present study supports a broader framework for examining the urethra as an interdependent multicellular tissue rather than an isolated muscular structure. The treatment-associated differences observed here involve cellular composition, inferred intercellular communication, and intrinsic transcriptional states. Because the principal single-cell comparisons are based on limited biological replication, they do not establish complete biological recovery or a definitive MAP-specific mechanism. Rather, they provide testable directions for future functional, histologic, and mechanistic validation.

### 3.2. Potential Multicellular Mechanisms and Translational Implications

This exploratory single-cell study extends SUI from a disorder of the sphincter alone to a failure of multicellular tissue repair [18]. MAP-treated samples showed differences in cellular composition and predicted communication pathways related to ECM and basement membrane organization, vascular support, cell adhesion, neural guidance, and synaptic structure, together with differences in progenitor-associated transcriptional states. These observations provide testable hypotheses that may help explain the functional and structural improvements previously observed after MAP treatment in animal models of SUI [14]. Importantly, these pathways were nominated by computational inference; the present study did not directly test signaling activity, progenitor function, or restoration of continence.

Urethral sphincter function depends on both muscle integrity and coordinated neuromuscular activation. Age-associated reduction in neuron-projection programs and neuromuscular junctions may contribute to persistent SUI. In this exploratory analysis, MAP-treated samples showed greater representation of RELN, SLIT, semaphorin, NCAM, NRXN, MPZ, glutamate-related signaling, and postsynaptic-organization programs, together with differences in ECM and cell-adhesion signaling. Increased collagen and laminin information flow should not be interpreted simply as promotion of fibrosis. Notably, GO analysis showed reduced persistent wound-healing and mesenchymal-development programs together with enhanced oxidative metabolism and stress adaptation, a combination more consistent with maturation of repair than with uncontrolled fibrosis. These are differences in transcript-derived predictions and enrichment terms, not demonstrations of changes in neural connectivity, oxidative function, matrix maturation, or tissue recovery. The apparent direction of enrichment cannot establish whether the underlying response is adaptive or maladaptive.

Vascular signaling may represent another important mechanism. ANGPT, VEGF, PECAM1, and CDH5-associated signaling showed greater representation in selected MAP comparisons, and endothelial and lymphatic endothelial cells occupied prominent positions in differential communication networks. Adequate perfusion is essential for progenitor activation, muscle metabolism, immune resolution, and matrix turnover. These observations raise the hypothesis that vascular-niche remodeling may contribute to MAP-associated urethral repair, which will require direct experimental validation. In the absence of ligand–receptor protein measurements, pathway perturbation, perfusion assays, or other functional evidence in these samples, the vascular-niche interpretation remains an untested mechanistic hypothesis.

Progenitor-associated transcriptional states provide a potential link to previous observations that MAP can influence resident muscle progenitor biology [9,14]. Krt15, Met, Sox9, Itga7, and Cd34 marked distinct epithelial, muscle, and stromal/vascular repair populations. The overall progenitor-associated signature in MAP-treated samples differed from BAPN and remained distinct from sham, consistent with treatment-associated differences in progenitor-related transcriptional states rather than demonstrated progenitor reactivation. Tissue repair depends not only on progenitor abundance but also on microenvironmental signals that regulate survival, differentiation, and integration. MAP-treated samples showed lower stress-related transcriptional activity in selected vascular and striated-muscle populations while retaining repair-associated remodeling programs in epithelial and stromal compartments [18]. The present data show cell-type-specific differences in a predefined 15-gene SASP-associated module across treatment groups, but they do not establish beneficial suppression or reprogramming. Expression of Krt15, Sox9, Itga7, Met, or Cd34, and enrichment of their associated modules, cannot establish progenitor activation, proliferation, differentiation, lineage contribution, or tissue regeneration; functional assays and lineage-resolved validation are necessary.

Other energy-based approaches, including vaginal laser therapy, have also been investigated for SUI, with randomized sham-controlled trials evaluating their clinical efficacy [19]. In contrast, MAP represents a nonthermal mechanical stimulation strategy that may influence tissue remodeling and regenerative responses through distinct mechanisms; however, direct comparative studies are required to determine differences in efficacy, durability, cellular targets, and safety. In addition, phalloidin and α-SMA staining are imperfect surrogates for cell-type-specific muscle mass, and the presented histological quantification does not separately establish smooth-muscle hyperplasia, striated-muscle fiber regeneration, or an increase in muscle-cell number. Direct assessment would require anatomical compartment-specific, validated muscle markers and quantitative stereological or morphometric measurements in a replicated cohort.

Future clinical studies of MAP should incorporate validated patient-reported outcome measures in addition to objective functional and anatomical endpoints. Instruments such as the King’s Health Questionnaire (KHQ) and the International Consultation on Incontinence Questionnaire-Short Form (ICIQ-SF) can provide complementary assessment of urinary-incontinence symptom burden, quality-of-life impact, and patient-perceived treatment benefit [20].

This study has important limitations. First, the transcriptomic cohort included only eight animals, with one animal in each age-by-treatment condition. Thus, the two animals within each treatment group differed in age, and the independent contributions of age and treatment cannot be resolved. The large number of cells obtained from each animal does not constitute independent biological replication, and cell-level statistical results should not be interpreted as treatment-level inference. Second, CellChat infers communication from transcript abundance and curated ligand-receptor databases and does not demonstrate protein-level signaling or physical interaction. Third, stem/progenitor and SASP classifications depend on prespecified gene-set definitions and should be regarded as operational transcriptional signatures rather than definitive lineage identities; lineage tracing and orthogonal validation are required. Fourth, the separate histological cohort provides tissue-level support for muscle-content differences, but the principal transcriptomic findings have not been independently validated by targeted qPCR, Western blotting, or immunohistochemistry. Finally, balanced downsampling may improve comparability but reduce detection of rare populations, and some sample-level preprocessing metrics were not available in sufficient detail for retrospective reconstruction. Accordingly, the transcriptomic results are presented as descriptive, hypothesis-generating observations to guide future studies with independent biological replication.

## 4. Materials and Methods

### 4.1. Animals and Experimental Design

All animal procedures were conducted in accordance with institutional guidelines and were approved by the Institutional Animal Care and Use Committee of the University of California, San Francisco. This scRNA-seq study included eight female Sprague-Dawley rats. Animals were randomly assigned to four groups: sham control, vaginal balloon dilation plus ovariectomy (VBDO), VBDO plus beta-aminopropionitrile (BAPN), and VBDO plus BAPN and MAP (MAP). Each group contained one 24-week-old and one 48-week-old animal, yielding a 4-treatment × 2-age experimental design (Table 1).

A separate histological cohort consisting of 16 additional 24-week-old female Sprague–Dawley rats was used for immunofluorescence and muscle-tissue quantification. These animals were randomly assigned to the same four experimental groups (Sham, VBDO, BAPN, and MAP) and were not included in the scRNA-seq cohort. The number of animals in each histological group was Sham, *n* = 4; VBDO, *n* = 4; BAPN, *n* = 4; and MAP, *n* = 4 (total *n* = 16).

Sham animals underwent anesthesia, vaginal catheter insertion, and surgical preparation without balloon dilation or ovariectomy. Simulated childbirth injury was induced using an 18-Fr Foley balloon (Bard Medical, C. R. Bard, Inc., Covington, GA, USA) filled with 4 mL of water and maintained under a 130-g load for 4 h; bilateral ovariectomy (OVX) was performed 1 week later [13,14]. Beginning at Week 2, animals in the BAPN group received BAPN (300 mg/kg, intraperitoneally, twice weekly) for 4 weeks. Animals in the MAP group received the same BAPN regimen together with MAP treatment applied to the pelvic floor/urethral region at an energy flux density of 0.033 mJ/mm^2^, 3 Hz, 500 pulses per session, twice weekly for 4 weeks [14]. After completion of the 4-week treatment period, animals underwent a 1-week washout. All experimental groups were followed to the same predefined Week 7 endpoint, at which urethral tissues were collected. Thus, the BAPN and MAP groups were time-matched for tissue collection. A detailed experimental timeline is provided in Figure 1a and Figure S1.

### 4.2. Single-Cell RNA Sequencing and Data Preprocessing

Fresh urethral tissue was enzymatically dissociated to generate single-cell suspensions. Libraries were constructed using the 10× Genomics Chromium Single Cell 3′ platform and sequenced on an Illumina NovaSeq instrument (Illumina, Inc., San Diego, CA, USA). Raw reads were aligned to the Rattus norvegicus reference genome using Cell Ranger. Cells with 500–9000 detected genes, fewer than 25,000 unique molecular identifiers, and less than 20% mitochondrial transcripts were retained. Doublets, low-quality droplets, and cells without reliable annotation were removed. Sample-level cell recovery and sequencing quality-control metrics are provided in Table S1.

Following quality control, 161,561 cells were available across the eight samples. To reduce disproportionate representation of samples with higher cell recovery, sample-level downsampling was subsequently performed, retaining 23,128 cells for balanced comparative downstream analyses. This balancing step was performed after QC and was not part of the QC exclusion process; it also did not increase the number of biological replicates. Subsequent analyses were performed in R 4.6.0 using Seurat 5.5.0 and SeuratObject 5.4.0. Seurat was used for normalization, variable-feature selection, integration, principal-component analysis, neighborhood construction, clustering, and UMAP visualization. Additional analyses used CellChat 2.2.0.9001 for inferred cell-cell communication and clusterProfiler 4.20.0 for functional enrichment. Full software and package-version information is provided in the Supplementary Materials. The numbers of post-QC cells and cells retained for downstream analysis are reported for each of the eight animals in Supplementary Table S1 (2693–2982 cells retained per animal). The final dataset size was determined by the sum of the sample-level subsets selected to achieve approximately comparable representation of individual animals, rather than by a further mitochondrial or gene-count exclusion threshold. This description does not specify an unverified random seed or sampling implementation.

### 4.3. Cell-Type Annotation and Differential Expression Analysis

Clusters were annotated using canonical markers and differentially expressed marker genes as described below. Reanalysis identified 19 annotated cell-type/state categories: ciliated epithelial, luminal urothelial, secretory epithelial, secretory/glandular epithelial, squamous epithelial, squamous/basal epithelial, tuft/chemosensory epithelial, neuroendocrine/chemosensory, epithelial/neural-like, neural/epithelial-like, endothelial, endothelial subtype, lymphatic endothelial, fibroblast, stromal/vascular-associated, immune, smooth muscle, striated muscle, and high-mitochondrial/stressed populations. For exploratory candidate-gene identification, cell-level expression differences were assessed using the Wilcoxon rank-sum test in Seurat. GO enrichment analysis was then performed using genes showing higher or lower expression in MAP-treated samples relative to BAPN samples. Because each age-by-treatment condition was represented by one animal, these cell-level adjusted *p*-values and downstream enrichment results were used only for hypothesis generation.

### 4.4. Stem/Progenitor and SASP Analyses

Progenitor-associated transcriptional states were assessed using prespecified lineage-specific multigene panels: epithelial (Krt5, Krt14, Krt15, Krt17, Krt19, Trp63, Sox2, Klf5, Itga6, and Itgb4), muscle (Pax7, Myf5, Itga7, Vcam1, Sdc4, Spry1, Met, and Cdh15), and stromal/vascular (Pdgfra, Cd34, Pi16, Dpp4, Col15a1, Ly6a, Pdgfrb, Cspg4, Mcam, and Rgs5). Single-cell module scores were calculated using Seurat AddModuleScore based on normalized RNA expression and expression-matched control genes. Continuous module scores were used for group-level descriptive comparisons and were interpreted as progenitor-associated transcriptional states rather than definitive lineage identities. Met was retained in the prespecified muscle-associated module used for scoring; Figure 3C and Figure S3B visualize its expression as separate descriptive analyses.

A predefined 15-gene SASP-associated panel comprising Il6, Il1a, Il1b, Ccl2, Cxcl1, Cxcl2, Cxcl3, Csf2, Csf3, Mmp3, Mmp9, Serpine1, Timp1, Tgfb1, and Icam1 was used. All 15 genes were detected in the scRNA-seq dataset. Single-cell SASP-associated module scores were calculated using Seurat AddModuleScore based on normalized RNA expression and expression-matched control genes. Module scores were subsequently averaged within each annotated cell type and treatment group for visualization. Because SASP programs are context- and lineage-dependent, these scores were interpreted as relative transcriptional signatures rather than as evidence of a uniform beneficial or detrimental senescence response.

### 4.5. Cell-Cell Communication Analysis

CellChat was used to infer ligand-receptor communication among major urethral cell populations and to compare MAP with sham, VBDO, and BAPN. Outcomes included the number and aggregate strength of predicted interactions, incoming and outgoing signaling patterns, and relative information flow across signaling families. Pathways of particular interest included ECM and basement membrane signaling (COLLAGEN, LAMININ, and FN1), angiogenic signaling (ANGPT and VEGF), adhesion and junctional signaling (CDH, JAM, NECTIN, CLDN, and PECAM1), developmental and regenerative signaling (WNT, ncWNT, NOTCH, EGF, FGF, and TGFβ), and neural/neuromuscular communication (AGRN, NCAM, NRXN, RELN, SLIT, semaphorins, Netrin, MPZ, and glutamate-related signaling). The evidence for greater muscle-associated tissue staining in MAP-treated urethras comes from the independent histological cohort (Figure 2c,d), not from treatment-split UMAPs; the present study does not establish increased muscle-cell numbers or de novo regeneration.

### 4.6. Immunofluorescence Staining

Tissues were fixed in cold 2% formaldehyde containing 0.002% saturated picric acid in 0.1 M phosphate buffer (pH 8.0) for 4 h, cryoprotected overnight in 30% sucrose, embedded in OCT compound, and stored at −70 °C. Cryosections (10 μm) were mounted on SuperFrost-Plus slides, treated with 0.3% H_2_O_2_ in methanol for 10 min, washed with PBS, and blocked for 30 min in 3% horse serum containing 0.3% Triton X-100. For smooth muscle staining, sections were incubated overnight at 4 °C with an anti-smooth muscle actin antibody (SMA; 1:1000, Abcam), followed by an Alexa Fluor 594-conjugated secondary antibody (1:500, Invitrogen). For F-actin visualization, cryosections were washed with PBS, permeabilized with 0.1% Triton X-100 for 5–10 min, and incubated with Alexa Fluor 488-conjugated phalloidin in PBS for 30–60 min at room temperature in the dark. Sections were then washed with PBS, counterstained with DAPI, mounted with antifade mounting medium, and imaged by fluorescence microscopy. Histological quantification was performed by an investigator blinded to the treatment group. Microscopic fields/sections were selected according to predefined criteria, and the same number of fields/sections was analyzed for all animals, with five fields/sections evaluated per animal. Measurements from these technical measurements were averaged within each animal to generate a single animal-level value for statistical analysis. For Figure 2d, ImageJ was used to measure α-SMA-positive (red) and phalloidin/F-actin-positive (green) staining areas within the selected urethral tissue region of interest (ROI). Muscle content (%) was calculated as [(red-positive area + green-positive area)/total tissue ROI area] × 100. This measurement reflects the fraction of tissue area represented by the summed positive staining areas, rather than fluorescence intensity or a direct count of muscle cells.

### 4.7. Statistical Analysis

The animal was considered the biological experimental unit. This was an exploratory single-cell study with one animal in each age-by-treatment condition. Cell-level tests were used to prioritize candidate genes and pathways rather than to infer population-level treatment efficacy. Accordingly, individual cells were not treated as independent biological replicates, and formal age-by-treatment interaction testing was not performed. For the independent histological cohort, each animal contributed one summarized value to the statistical analysis (*n* = 4 per group); sections and microscopic fields were treated as technical measurements. Muscle-associated staining measurements were compared among the four groups using one-way ANOVA followed by Tukey’s multiple-comparisons test and are presented as mean ± SEM, as shown in Figure 2d. The small sample size limits the reliability of distributional-assumption assessments and precision of effect estimates; accordingly, histological comparisons are interpreted cautiously and require replication. Specifically, adjusted *p*-values from Seurat/Wilcoxon transcriptomic comparisons reflect exploratory cell-level testing and should not be interpreted as population-level evidence of treatment efficacy.

## 5. Conclusions

MAP treatment was associated with transcriptomic differences in the urethral microenvironment following simulated childbirth injury. The exploratory analyses identified treatment-associated differences in epithelial, vascular, immune, stromal, and muscle cell representation; CellChat-inferred matrix-, adhesion-, angiogenic-, neural-, and neuromuscular communication patterns; and progenitor-associated, SASP-associated, and injury/stress-related transcriptional states. A separate histological cohort showed higher urethral muscle content in MAP-treated animals than in injured comparators. These findings are hypothesis-generating and do not establish treatment efficacy or causal mechanisms. Independent biological replication, prospectively documented experimental procedures, functional testing, orthogonal molecular validation, and targeted mechanistic studies are required to confirm the observations and determine their biological significance. The small histological cohort and the descriptive, non-replicated age-by-treatment transcriptomic comparisons preclude definitive claims of regeneration or improved function based on these readouts alone.

## Figures and Tables

**Table 1:** Experimental design of the single-cell RNA-sequencing cohort (*n* = 8).

Treatment Group	24 Weeks	48 Weeks	Total
Sham	1	1	2
VBDO	1	1	2
BAPN	1	1	2
MAP	1	1	2
Total	4	4	8

## Data Availability

The single-cell RNA-sequencing data supporting this study are available in the NCBI Gene Expression Omnibus under accession number GSE348358. A secure reviewer token (cnwdyiwclpifteb) has been generated to allow confidential access during peer review. The GEO record is currently in private status and is scheduled for public release on 6 January 2027.

## Supplementary Materials

The following supporting information can be downloaded at: https://www.mdpi.com/article/10.3390/ijms27219833/s1.
