# Supplementary material for: Single-Cell Transcriptomic Analysis of Rat Urethral Tissue Following Simulated Childbirth Injury and Microenergy Acoustic Pulse Therapy
Source: Int J Mol Sci. 2026 Nov 3;27(21):9833. doi: 10.3390/ijms27219833 (PMC13763710; doi:10.3390/ijms27219833)
Supplement: Supplementary file 1 [file ijms-27-09833-s001.zip › ijms-4640936-supplementary.pdf]

Single-Cell Transcriptomic Analysis of Rat Urethral Tissue Following Simulated Childbirth Injury and Microenergy Acoustic Pulse Therapy

Guiting Lin [1], Liangyu Zhao [1,2], Emily Xing [1], Tian Bai [1], Hao Li [1,3], Yuan Tang [1,4], Thomas Cao [1], Guifang Wang [1], Yuxin Tang [1,2] and Tom F. Lue [1,*]

Bioinformatic analyses were performed using R version 4.6.0. Major packages included Seurat (v5.5.0), SeuratObject (v5.4.0), CellChat (v2.2.0.9001), clusterProfiler (v4.20.0), ComplexHeatmap (v2.28.0), dplyr (v1.2.1), ggplot2 (v4.0.3), tidyr (v1.3.2), and igraph (v2.3.2).

Supplementary Figures

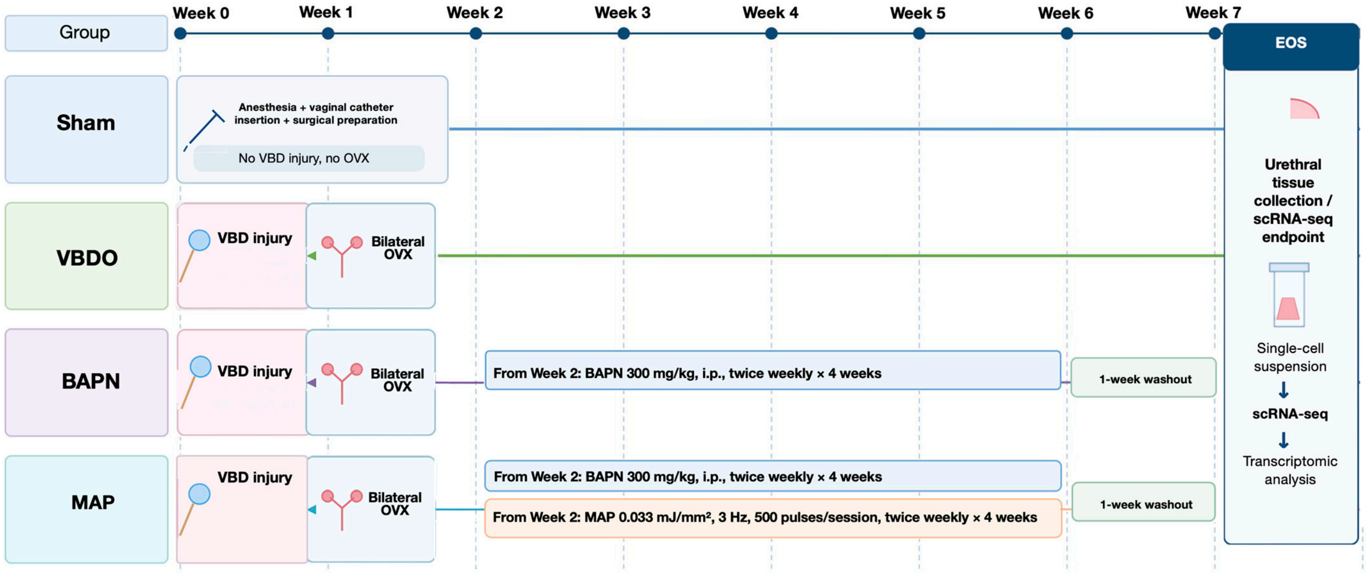

Figure S1. Schematic overview of the experimental design and treatment timeline. Female rats were assigned to Sham, VBDO, BAPN, and MAP groups. VBD was performed at Week 0 in the VBDO, BAPN, and MAP groups, followed by bilateral OVX at Week 1. Beginning at Week 2, the BAPN group received BAPN (300 mg/kg, i.p.) twice weekly for 4 weeks, whereas the MAP group received the same BAPN regimen together with MAP treatment (0.033 mJ/mm², 3 Hz, 500 pulses/session, twice weekly for 4 weeks). Sham animals underwent anesthesia, vaginal catheter insertion, and surgical preparation without VBD or OVX. After a 1-week washout period, animals were euthanized at Week 7, and urethral tissues were collected for single-cell suspension preparation, scRNA-seq, and transcriptomic analysis. EOS: end of study.

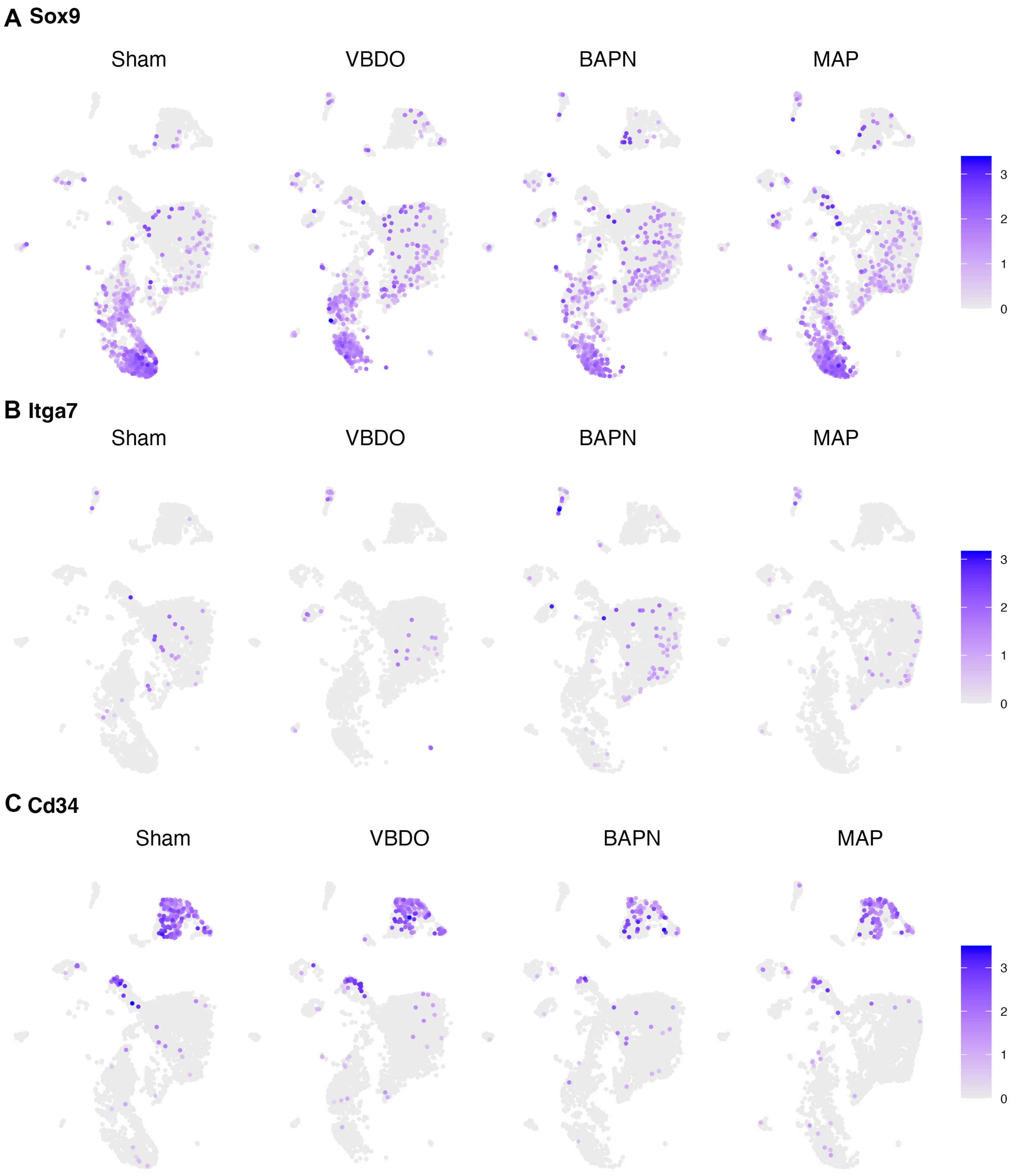

Figure S2. Treatment-specific expression patterns of Sox9, Itga7, and Cd34. (A) Treatment-split UMAP feature plots showing Sox9 expression in Sham, VBDO, VBDO+BAPN, and VBDO+BAPN+MAP groups. (B) Treatment-split UMAP feature plots showing Itga7 expression across the four experimental conditions. (C) Treatment-split UMAP feature plots showing Cd34 expression across the four experimental conditions. For each gene, identical UMAP coordinates and a common within-gene expression scale were used across treatment groups. Because expression of these genes was

relatively sparse and lineage-restricted, the plots are intended to provide descriptive visualization of their cellular distribution rather than formal treatment-level statistical inference. For visualization, cells from the 24- and 48-week animals were displayed together within each treatment group; these plots do not constitute independent treatment-level biological replication. Similar marker-gene localization across conditions is not a measurement of muscle-cell abundance, and Itga7 expression is sparse.

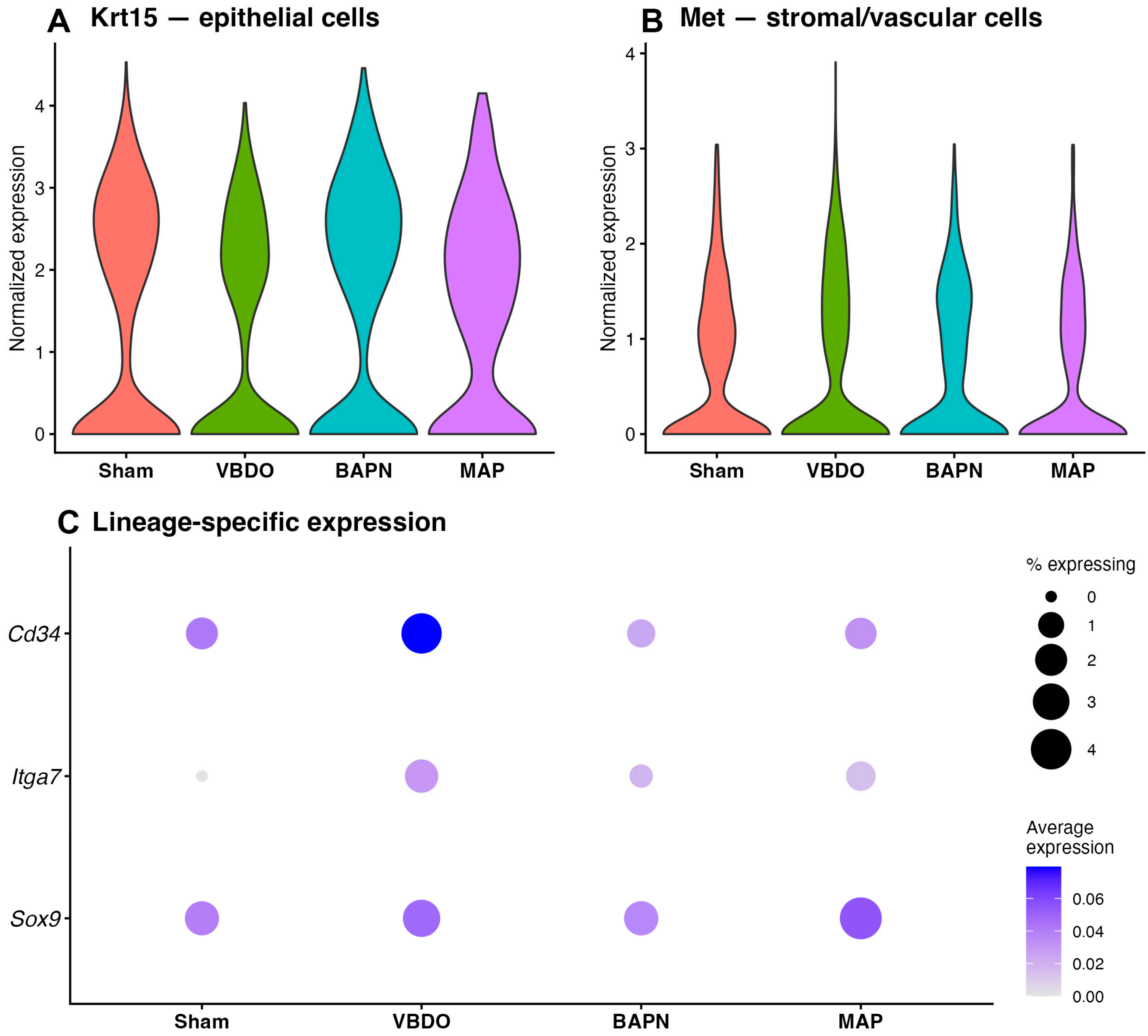

Figure S3. Lineage-specific expression of selected treatment-associated genes. (A) Violin plots showing Krt15 expression in epithelial cell populations across Sham, VBDO, VBDO+BAPN, and VBDO+BAPN+MAP conditions. (B) Violin plots descriptively visualizing detectable Met expression within stromal/vascular cell populations across the four experimental conditions. (C) Lineage-specific dot plots showing Sox9 expression in epithelial cells, Itga7 expression in muscle cells, and Cd34 expression in stromal/vascular cells. Dot size represents the percentage of cells with detectable expression, whereas color intensity represents average normalized expression. These plots are presented as descriptive cell-level visualizations and are not interpreted as formal treatment-level statistical comparisons. For visualization, cells from the 24- and 48-week animals were displayed together within each treatment group; these plots do not constitute

independent treatment-level biological replication. These displays are descriptive, not evidence of expansion of progenitors or muscle-cell number.

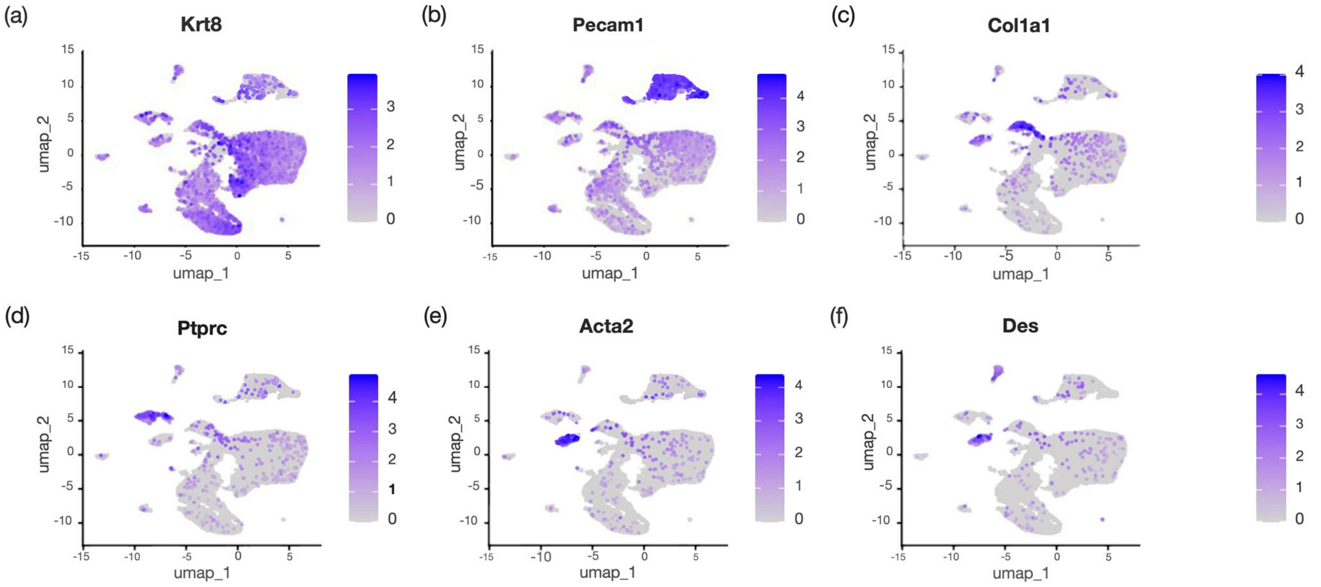

Figure S4. Cell-type annotation and representative marker-gene expression in the rat urethral single-cell RNA-sequencing dataset. UMAP feature plots showing the expression of representative marker genes used for major cell-type annotation: (a) Krt8, epithelial cells; (b) Pecam1, endothelial cells; (c) Col1a1, fibroblasts/stromal cells; (d) Ptprc, immune cells; (e) Acta2, smooth muscle cells; and (f) Des, muscle-lineage cells. Color intensity represents relative gene expression levels, with darker blue indicating higher expression. Feature plots share identical UMAP coordinates and orientation with Figure S2A to facilitate direct identification of expressing cell populations.

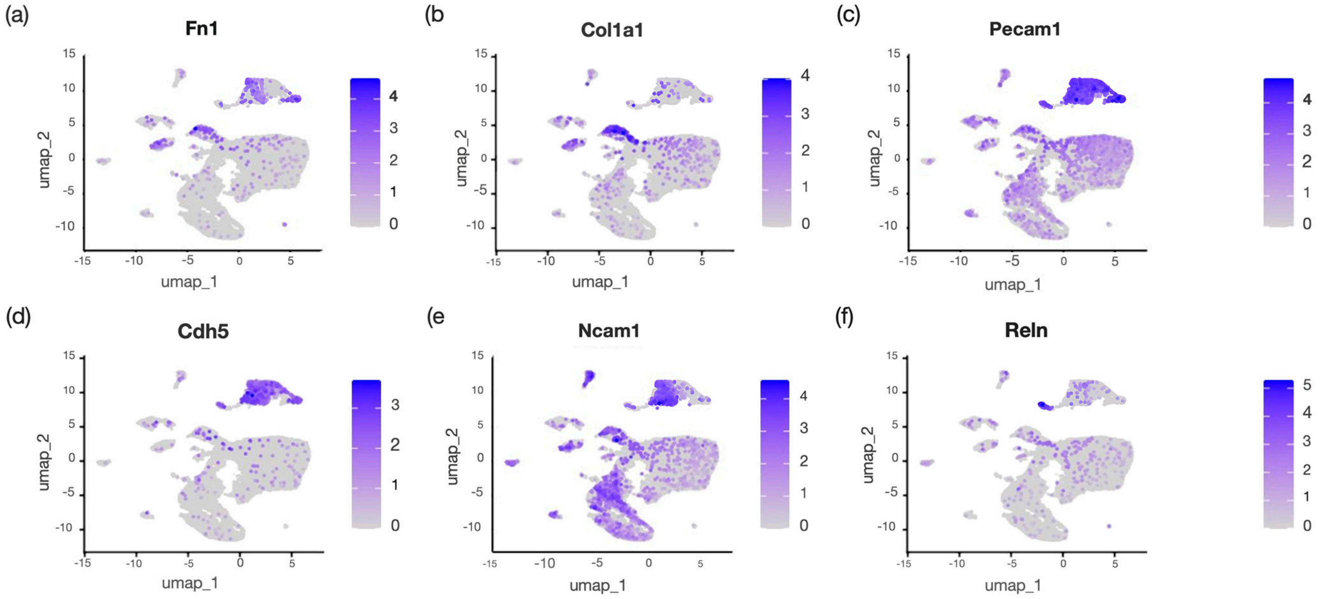

Figure S5. Cell-type localization of representative pathway-associated genes in the rat urethral single-cell RNA-sequencing dataset. UMAP feature plots showing the distribution of representative pathway-associated genes across identified cell populations: (a) Fn1, (b) Col1a1, (c) Pecam1, (d) Cdh5, (e) Ncam1, and (f) Reln. Color intensity indicates relative gene expression, with darker blue representing higher expression levels. UMAP coordinates and orientations are identical to those in Figure S2A to allow direct cell-type cross-referencing.

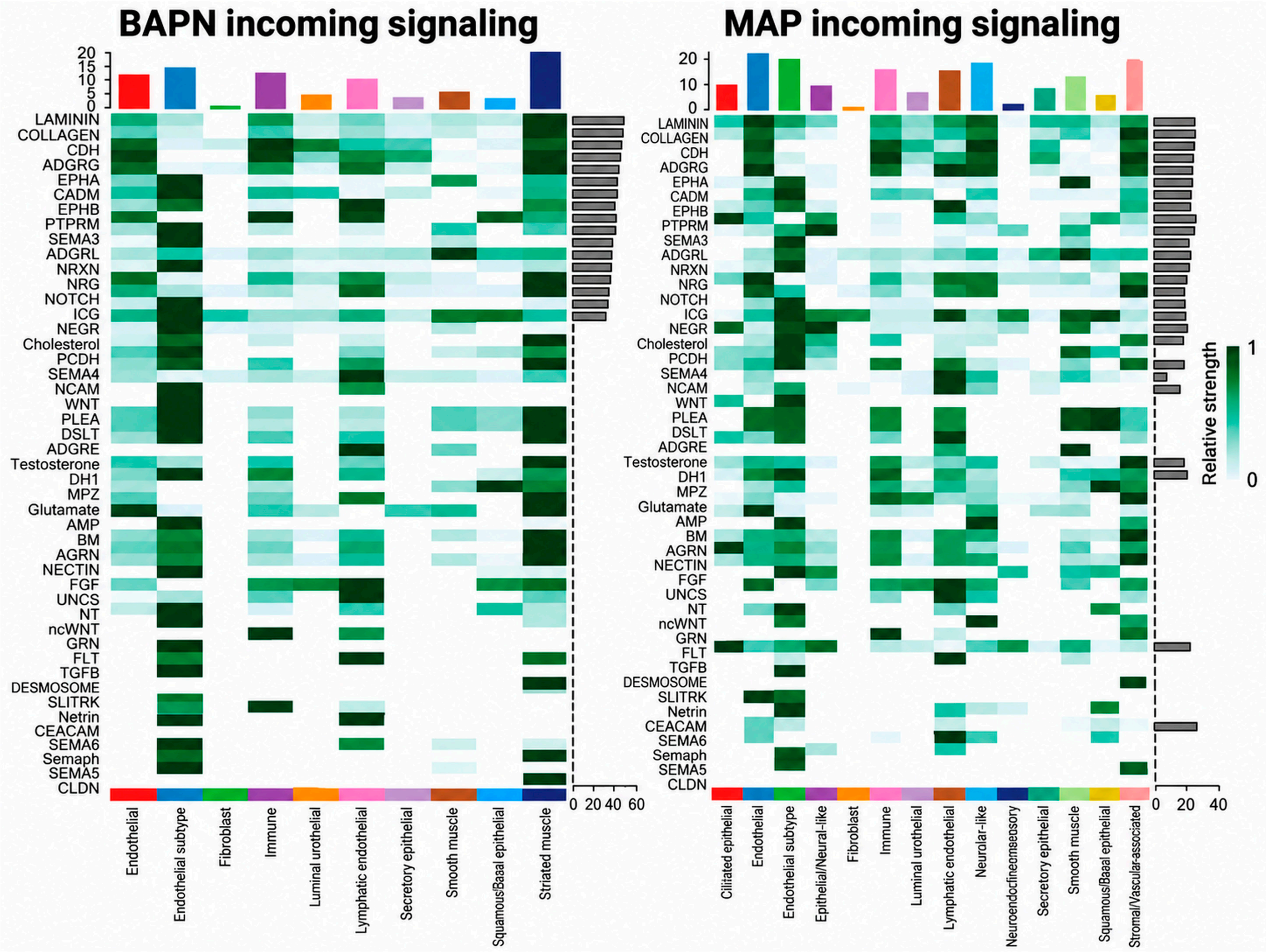

Figure S6. Enlarged version of main-text Figure 5B. Heatmaps of incoming signaling activity, reflecting receptor-associated signaling received by each cell population in the BAPN and MAP groups. Rows represent signaling pathways and columns represent recipient cell types. Darker green indicates greater relative incoming signaling activity.

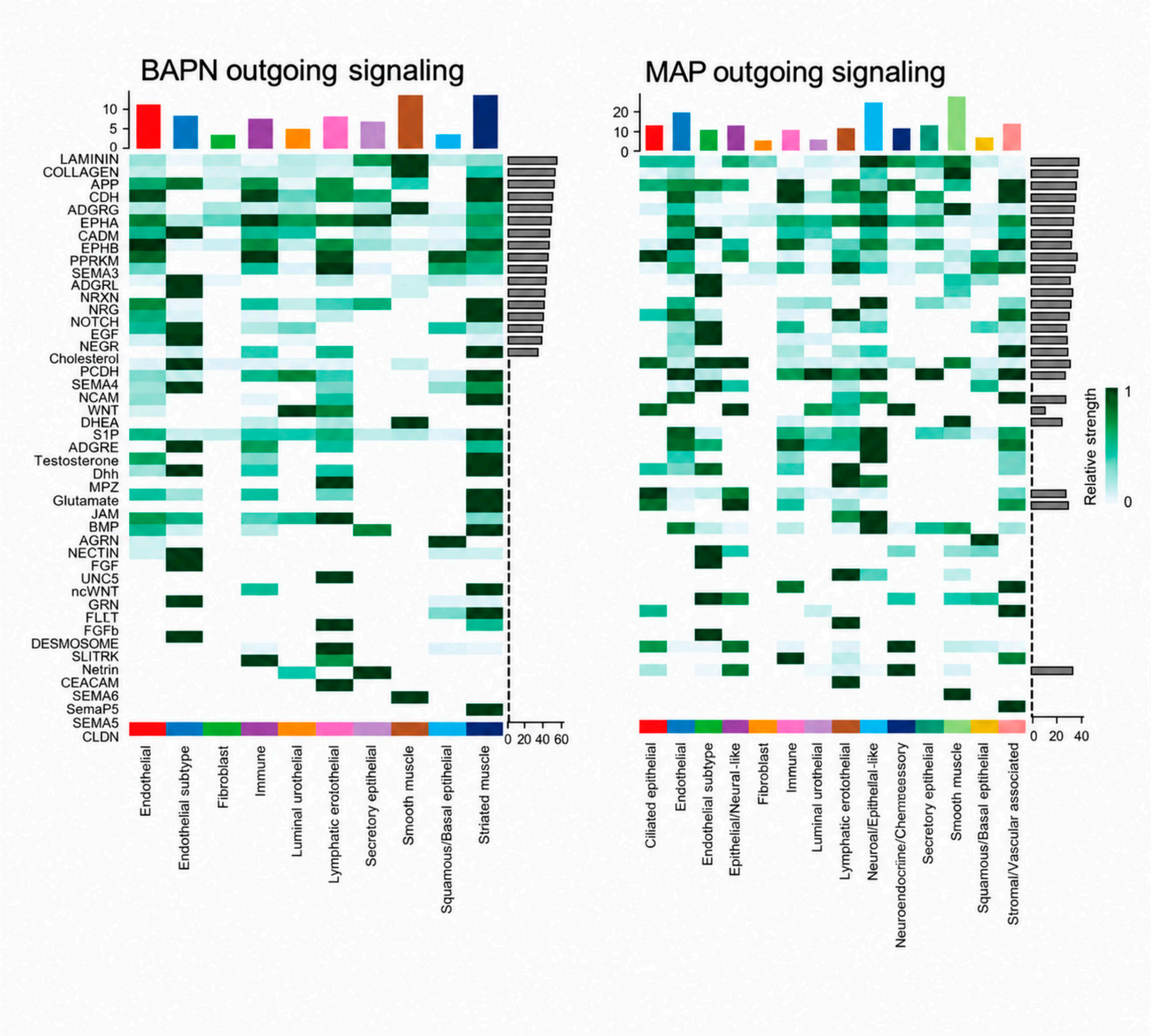

Figure S7. Enlarged version of main-text Figure 5C. Heatmaps of outgoing signaling activity, reflecting ligand-associated signaling transmitted by each cell population in the BAPN and MAP groups. Rows represent signaling pathways and columns represent sender cell types. Darker green indicates greater relative outgoing signaling activity.

Supplementary Tables

Table S1. Sample-level cell recovery and sequencing quality-control metrics.

Sample	Age (wks)	Starting cells	Post-QC cells before downsampling	Cells retained for downstream analysis	Median genes/cell	Median UMI/cell	Median mitochondrial reads (%)
BAPN 24	24	19,133	17,708	2,952	1,489	2,161.5	0.52
BAPN 48	48	22,179	20,163	2,693	2,795	5,568.0	0.74
MAP 24	24	33,544	32,398	2,917	2,854	5,737.5	0.34
MAP 48	48	19,498	17,997	2,889	2,313	4,372.0	0.48
VBDO 24	24	17,914	16,051	2,787	1,890	3,099.0	0.24
VBDO 48	48	15,939	14,961	2,967	1,919	3,280.0	0.42
sham 24	24	35,662	33,017	2,941	1,519	2,194.0	0.66
sham 48	48	9,787	9,266	2,982	3,034	7,329.0	0.37

Note: "Post-QC cells before downsampling" indicates the number of cells retained after the initial quality-control and filtering procedures and before sample-level balancing. Across the eight samples, 161,561 cells were available at this stage. To reduce unequal representation resulting from differences in cell recovery among samples, sample-level downsampling was subsequently performed, retaining 23,128 cells for comparative downstream analyses. Downsampling was performed as a balancing step and did not increase the number of biological replicates.
